# Bi_2_O_3_ and g-C_3_N_4_ quantum dot modified anatase TiO_2_ heterojunction system for degradation of dyes under sunlight irradiation

**DOI:** 10.1039/c9ra07424d

**Published:** 2020-01-07

**Authors:** Weidong Peng, Chun Yang, Jiang Yu

**Affiliations:** College of Architecture and Environment, Sichuan University Chengdu 610065 China yuj@scu.edu.cn; Institute of New Energy and Low Carbon Technology, Sichuan University Chengdu 610065 China; College of Chemistry and Materials Science, Sichuan Normal University Chengdu 610068 China; Computational Visualization and Virtual Reality Key Laboratory of Sichuan Province Chengdu 610068 China

## Abstract

A facile and feasible method was successfully utilized to incorporate Bi_2_O_3_ and g-C_3_N_4_ quantum dots on TiO_2_ surface to synthesize a novel composite g-C_3_N_4_/TiO_2_/Bi_2_O_3_. The photocatalytic activity of the composite g-C_3_N_4_/TiO_2_/Bi_2_O_3_ for degradation of dyes under sunlight and UV light irradiation was evaluated. It possessed the higher photocatalytic performance than that of pristine TiO_2_ or g-C_3_N_4_ under the same conditions. Under sunlight irradiation, the reaction rate constants of the g-C_3_N_4_/TiO_2_/Bi_2_O_3_ was about 4.2 times and 3.3 times higher than that of TiO_2_ and g-C_3_N_4_, respectively. The promising photocatalytic performance was attributed to the broader light absorption range and efficient separation of photoinduced carriers. Moreover, based on the TEM, XPS, XRD, UV-vis spectrum, radicals scavenging test and Mott–Schottky analysis systematic mechanism for photodegradation process was proposed. This work provides a promising strategy for the modification of TiO_2_-based semiconductors by incorporating different quantum dots and promoting the efficiency of the photocatalysts in practical application.

## Abstract 2

A facile and feasible method was successfully utilized to incorporate Bi_2_O_3_ and g-C_3_N_4_ quantum dots on TiO_2_ surface to synthesize a novel composite g-C_3_N_4_/TiO_2_/Bi_2_O_3_.

## Graphical abstract

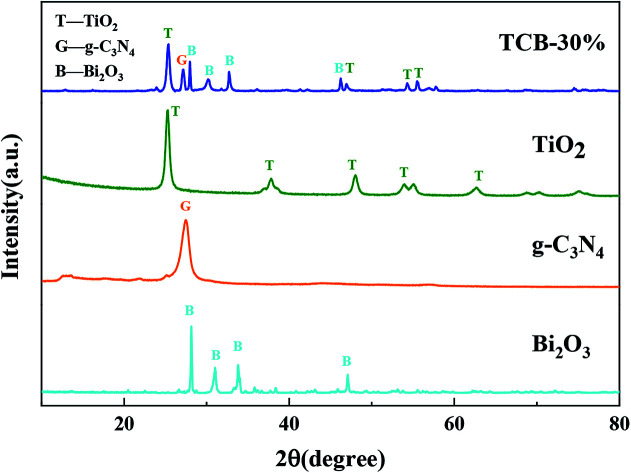

## 1 Introduction

At present, environmental contamination and energy shortage are the most serious problems on our planet. Hence, there is an urgent need for the development of an environmental, sustainable, economical and energy-saving technology for contamination control. Semiconductor photocatalysis provides an optimal solution for the problems with the potential that the clean solar energy can be harnessed for decomposition of pollutants.^1^ To date, TiO_2_ as the photocatalyst has drawn significant attention and been widely studied because of its desirable properties including high reactivity, chemical stability, nontoxicity, and cheap price.^2–7^ Nevertheless, the application of TiO_2_-based photocatalysts is hindered due to its inefficient utilization of solar energy, poor charge separation, and low adsorption of organic contaminants on the surface. Besides these intrinsic drawbacks, the complicated process of synthesis is another key factor to impede its practical application.^8^

To avoid the above limitation, various modification strategies have been carried out, such as dye-sensitization, metal or non-metal doping, and transition metal doping, *etc.* Particularly, compositing TiO_2_ with different semiconductor into heterojunction is an efficient method to enhance the photocatalytic activity.^8^ Since the heterojunction structure would decrease the recombination of electron/hole by separating the charge carriers to different semiconductors surface due to the different potential barriers. These heterojunctions-based semiconductors exhibit advanced efficiency for degrading pollutants.

Presently, considerable attention has been paid to graphitic carbon nitride (g-C_3_N_4_), which is a stable kind of polymers with a layered structure like graphene. Particularly, it possesses the strong ability to harvest solar energy due to its narrow band gap (*E*_g_ = 2.7 eV). Nevertheless, the quantum efficiency of pure g-C_3_N_4_ is low because of the high recombination ratio of photogenerated electron/hole pair.^9^ Combining the TiO_2_ with g-C_3_N_4_ in heterojunction is considered as an efficient method which can improve the absorption of visible light and the separation of photogenerated pairs. Yu *et al.*^10^ synthesized g-C_3_N_4_–TiO_2_ composites by calcination process using P25 and urea, achieving the high photoactivity for the degradation of HCHO. Lei *et al.*^11^ reported the synthesis of g-C_3_N_4_/TiO_2_ photocatalyst *via* simple calcination using cyanamide and anatase TiO_2_ as precursor. The g-C_3_N_4_/TiO_2_ photocatalyst exhibits a promising progress in degradation of the dye Acid Orange 7 under both visible and UV light. The g-C_3_N_4_–TiO_2_ composites with prominent photoactivity for the degradation of phenol under UV light was synthesized by Colo'n *et al.*^12^ by impregnation. Zhang *et al.*^13^ found that well-dispersed TiO_2_ nanocrystals with (001) facets prepared *in situ* on g-C_3_N_4_ through a solvothermal method exhibit higher efficiency for photocatalytic degradation of phenol as compared to pure g-C_3_N_4_ and TiO_2_. Wu *et al.*^14^ prepared nanosheets TiO_2_/g-C_3_N_4_ composite by solvothermal method, which exhibits a significant improvement in photodegradation towards methylene blue under visible light irradiation than pristine g-C_3_N_4_ and TiO_2_.

Among strategies of constructing heterojunction, synthesis of p–n junction is considered as an effective method to facilitate the photoactivity by inducing the separation of photogenerated carries due to the existence of an internal electric field. Bismuth oxide (Bi_2_O_3_), as a p-type semiconductor, has been extensively studied due to its suitable band gap (*E*_g_ = 2.8 eV) and visible light driven catalytic activity. Among the six different polymorphic phases of Bi_2_O_3_, β-Bi_2_O_3_ has prominent advantages for degradation of pollutants. Since TiO_2_ is a n-type semiconductor, it is feasible to combine TiO_2_ with β-Bi_2_O_3_, by which catalytic efficiency can be notably enhanced.^15–18^ Recently, much efforts have been made to synthesize TiO_2_/Bi_2_O_3_ heterojunction. Various methods have been employed such as: pulse electro-deposition,^19^ hydrothermal, sol–gel, and coprecipitation.^20–26^ However, the complexity of these methods limits the application of photocatalysis.

Instead of above strategies, researchers have succeeded in sensitizing TiO_2_ by modifying quantum dots (QDs) of low-band gap materials such as g-C_3_N_4_, CdS, CdSe, CdTe, and Bi-based materials, which can absorb light in the visible region. QDs can match the solar spectrum better due to the particle size effect. Additionally, QDs are recently reported to generate multiple excitons, which can improve the photocatalysis efficiency.^27–30^ Jiao *et al.*^31^ firstly prepared Bi_2_O_3_ quantum dots decorated TiO_2_ with exposed {001} facets on graphene sheets. Size-controllable were *in situ* synthesized on TiO_2_ nanotube arrays and high activity in synergetic H_2_ evolution and organics degradation.^32^

In this study, we firstly use a simple ball-milling/calcination method to synthesize a g-C_3_N_4_/TiO_2_/Bi_2_O_3_ heterojunction system which distinctly exhibit a promising synergetic improvement effect. The different properties of the catalyst are evaluated by the degradation of simulated dyeing wastewater under UV light and direct sunlight irradiation. While the different properties are confirmed by TEM, XPS, XRD and UV-vis diffuse reflection spectra. A tentative mechanism for photocatalytic degradation of RhB by the composite g-C_3_N_4_/TiO_2_/Bi_2_O_3_ is proposed.

## 2 Materials and methods

### 2.1 Catalysts synthesis

#### 2.1.1. Chemicals and reagents

Double distilled water was used throughout the experiment. The catalyst precursor used in this experiment including titanium dioxide (TiO_2_, anatase, 99.8%; Shanghai Aladdin Biochemical Technology Co., Ltd), melamine (98%; Shanghai Aladdin Biochemical Technology Co., Ltd), bismuth(iii) nitrate-pentahydrate (Bi(NO_3_)_3_·5H_2_O, 98%; Shanghai Aladdin Biochemical Technology Co., Ltd). All the reagents utilized were of analytical grade and without further purification.

#### 2.1.2 Methods

##### 1. Synthesis of pure g-C_3_N_4_

5 g of melamine were added into crucible with a cover then heated at 520 °C under air atmosphere for 2 h with a heating rate of 5 °C min^−1^. After cooling to the room temperature naturally, the resulting yellow bulks were collected and ball-milled into powders for further evaluation.

##### 2. Synthesis of pure Bi_2_O_3_

3 g Bi(NO_3_)_3_·5H_2_O were added into crucible with a cover then heated at 520 °C under air atmosphere for 2 h with a heating rate of 5 °C min^−1^. After cooling to the room temperature naturally, the resulting bulks were collected and ball-milled into powders for further evaluation.

##### 3. Synthesis of g-C_3_N_4_/TiO_2_/Bi_2_O_3_

The g-C_3_N_4_/TiO_2_/Bi_2_O_3_ catalysts were prepared by the processes of ball-milling and calcination. Firstly, 400 mg of anatase TiO_2_ powders and 600 mg of melamine were added into the agate ball milling tank, followed by the process of ball milling for 2 h with a ball powder ratio of 10 : 1, at a speed of 400 rpm in a planetary ball mill. Then, a certain amount of Bi(NO_3_)_3_·5H_2_O were added into the agate ball milling tank which was filled with TiO_2_ and melamine. The powders mixed by ball milling for 2 h with a ball powder ratio of 10 : 1, at a speed of 400 rpm in a planetary ball mill. Subsequently, the mixed powder was placed into a crucible with a cover then heated at 520 °C under air atmosphere for 2 h with a heating rate of 5 °C min^−1^. After cooling to the room temperature naturally, the resulting yellow bulks were collected and ball-milled into powders for further evaluation. The obtained powers was named as TCB-*x*, where the *x* refers to the weight percentage of Bi(NO_3_)_3_·5H_2_O to the weight of TiO_2_ and melamine.

### 2.2 Characterization

The crystal phases was characterized by X-ray diffraction (XRD, Bruker D8 ADVANCE A25X) with a Cu Kα radiation with a diffraction angle between 10–80°

Transmission electron microscopy (TEM) and high-resolution transmission electron microscopy (HRTEM) were measured by Tecnai G2 F20 S-TWIN (200 kV). The samples were dispersed in ethanol and dropped on copper grids. Chemical composition and valence band (VB) were observed by X-ray photoelectron spectroscopy (XPS, Escalab 250Xi) using a monochromatic micro-focused Al Kα (1486. 6 eV) source. UV-vis diffuse reflectance spectroscopy (UV-vis DRS) absorption spectra of the wavelength between 200 nm and 800 nm were carried out at a Shimadzu UV-3600 spectrophotometer using BaSO_4_ as a reference.

The electrochemical studies were conducted on an electrochemical workstation (CHI660C, CH Instrument Corp, Shanghai), which used catalyst-deposited FTO glass as working electrode, Pt as the counter electrode and Ag/AgCl as the reference electrode. Meanwhile, 0.5 M Na_2_SO_4_ was served as the electrolyte solution. The working electrode was prepared as follows: 20 mg PEG-600 and 10 mg pure TiO_2_ and Bi_2_O_3_ were dispersed in 1 mL ethanol and ultrasonically scattered for 1 h. Then, the suspension above was added onto the FTO glass (1 × 1 cm) and evaporated to dry. The Mott–Schottky measurement was performed at frequency of 1000 Hz.

### 2.3 Photocatalytic activity test

The aqueous Rhodamine B (RhB), methylene blue (MB) and methyl orange (MO) dyes were used as model organic contaminants to evaluate the photocatalytic activities of the synthesized catalysts. Degradation experiments were carried out under UV and direct solar light irradiation. A high-pressure 300 W mercury lamp with the radiation of 365 nm was used as the UV light source. To study the photocatalytic activity of the samples under visible light, the photoreactor were directly exposed to sunlight. And the illumination intensity was measured by solar power meter (TES-1333R). 50 mg catalyst powders were suspended in 50 mL aqueous organic dye solution and the dyes concentration was 20 mg L^−1^. To reach adsorption–desorption equilibrium, the solution was stirred for 60 min in the dark. At given time intervals, 4 mL aliquots were taken and centrifuged at 10 000 rpm for 10 min to remove the particles. Then, the concentration of organic dyes in filtrates was analyzed using an Alpha-1506 UV-vis spectroscopy (Shanghai Lab-Spectrum Instruments Co. Ltd, China) at the wavelength of 554 nm, 664 nm, 465 nm for RhB, MB, MO, respectively. The experiments were repeated 3 times.

## 3 Result and discussion

### 3.1 XRD characterization

**Fig. 1:**
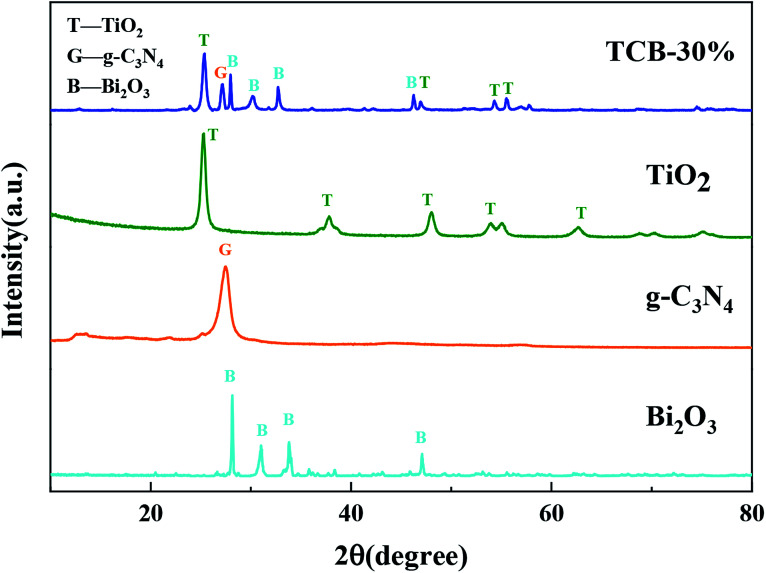
XRD pattern of TCB-30%, TiO_2_, g-C_3_N_4_ and Bi_2_O_3_.

Fig. 1 shows the XRD patterns of TCB-30%, pristine TiO_2_, g-C_3_N_4_ and Bi_2_O_3_. In TCB-30% patterns, the diffraction peaks at 25.3°, 37.9°, 48.0°, 53.9° could be well indexed to the (101), (004), (200) and (105) planes of the anatase phase TiO_2_.^23,33^ There are four peaks at 27.9°, 31.8°, 32.8°, 46.3°, corresponding to the (211), (002), (220), and (222) plane of pristine Bi_2_O_3_.^26,34,35^ The characteristic peaks of g-C_3_N_4_ at 27.5° (ref. 27 and 36) was also detected in TCB-30% patterns. The diffraction peaks with relatively low intensity indicated the small sized Bi_2_O_3_ and TiO_2_ quantum dots, in consistent with the results of TEM. The well crystallized diffraction peaks detected in TCB-30% patterns suggested the well crystalline structure of anatase TiO_2_, g-C_3_N_4_ and Bi_2_O_3_, without the generation of other crystal structure.

### 3.2 Microstructure analysis

**Fig. 2:**
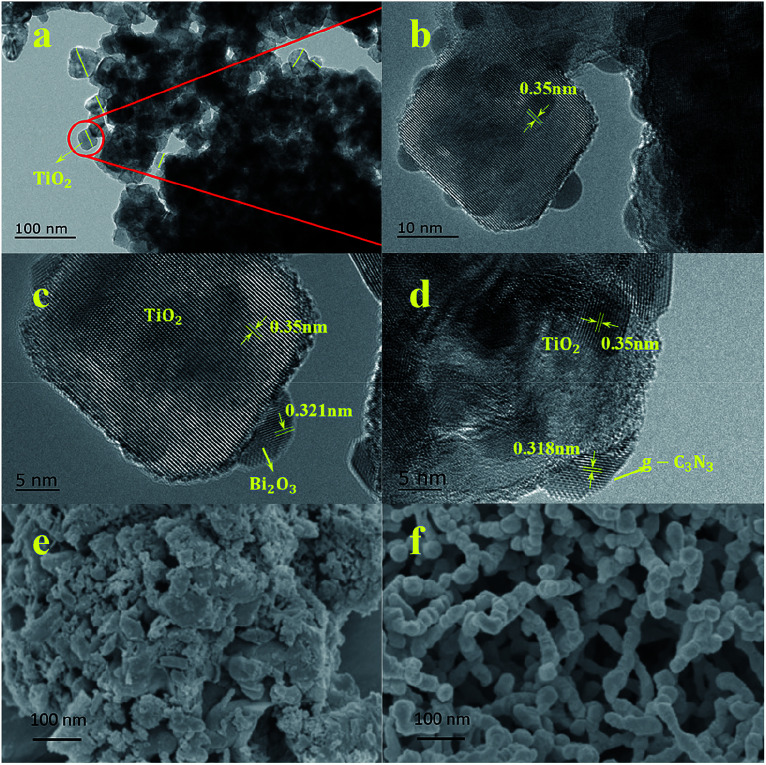
(a) Selected TEM figure of TCB-30%, (b, c and d) HRTEM figures of TCB-30%, (e) SEM figure of pure g-C_3_N_4_, (f) SEM figure of pure Bi_2_O_3_.

The morphology of the prepared catalysts was characterized by TEM and high-resolution. TEM image Fig. 2b, c and d are enlarged views of Fig. 2a. As shown in Fig. 2a, TiO_2_ particles have a spherical structure with an average size between 30–60 nm, which was in well accordance with the crystallite size obtained from Scherrer equation. Bi_2_O_3_ and g-C_3_N_4_ showed a much smaller size below 10 nm, and intimately cover on the surface of TiO_2_ particles, which intensively increase the specific surface area of TiO_2_.

Clear lattice fringes for the identification of crystallographic spacing could be observed in Fig. 2c, and the lattice spacing of 0.350 nm and 0.321 nm corresponded well with (101) phase of TiO_2_ and (221) phase of Bi_2_O_3_,^15–17^ respectively. In Fig. 2d, the lattice spacing of 0.318 nm matched the (002) crystal phase of g-C_3_N_4_.^37–39^

Thus, these TEM images indicated the interaction of g-C_3_N_4_/TiO_2_/TiO_2_ heterojunction composite. The g-C_3_N_4_ and Bi_2_O_3_ located on the surface of TiO_2_ as quantum dots. In Fig. 2e, the pure g-C_3_N_4_ showed aggregated morphologies, which were comprised of block-based flakiness. Fig. 2f displayed a spheroidal structure of Bi_2_O_3_. It was obvious that the process of ball-milling played a significant role in synthesizing g-C_3_N_4_ and Bi_2_O_3_ quantum dots on TiO_2_ surface.

### 3.3 Chemical compositions

**Fig. 3:**
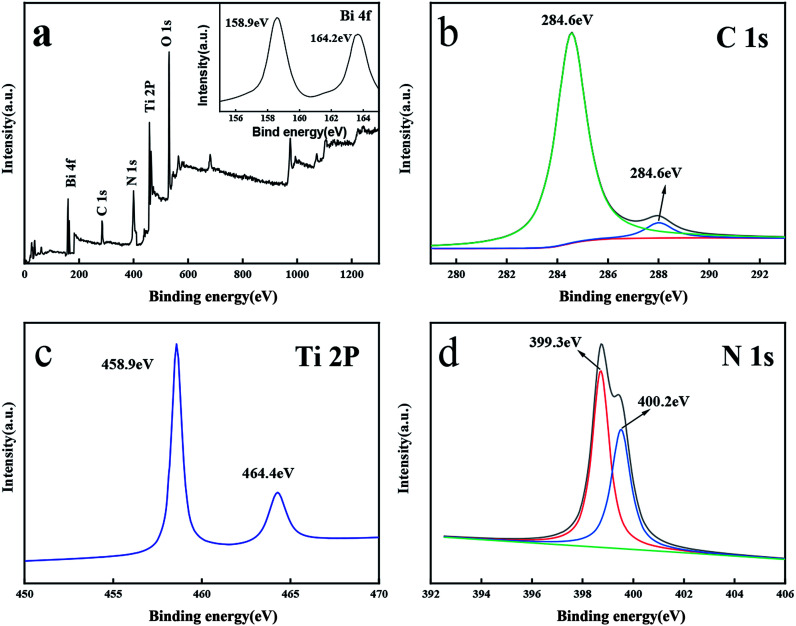
XPS spectrum of (a) TCB-30%, (b) C 1s, (c) Ti 2P, (d) N 1s.

Fig. 3 showed the full survey and high-resolution spectra for the Ti 2p, O 1s, C 1s, N 1s and Bi 4f region. Fig. 3b depicts the C 1s of the prepared. The peak centered at 284.6 eV could be assigned to the adventitious carbon while the peak at 287.5 eV belongs to the N–C=N group of the g-C_3_N_4_.^40–42^Fig. 3d showed N 1s spectrum of catalyst. The N 1s peak was fitted into two peaks at 399.3 eV and 400.2 eV, which were the sp^2^-hybridized nitrogen (C=N–C) and tertiary nitrogen (N-3), respectively.^40–42^ Furthermore, two fitted peaks of Ti 2p3/2 and Ti 2p1/2 at 458.9 eV and 464.4 eV suggested the presence of Ti(iv).^27,36,43^ In Fig. 3a, two fitted peaks at 158.9 eV and 164.3 eV belonged to Bi 4f5/2 and Bi 4f7/2, suggesting the existence of Bi_2_O_3_ in the catalyst.^24,34,35^ These results suggested that the composite TCB-30% consisted of g-C_3_N_4_, anatase TiO_2_ and Bi_2_O_3_.

### 3.4 UV-vis DRS analyses

**Fig. 4:**
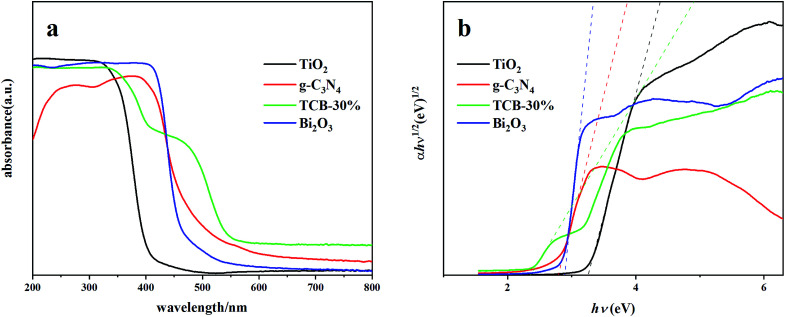
(a) UV-vis DRS spectra and (b) optical bandgap of the TCB-30%, TiO_2_, g-C_3_N_4_ and Bi_2_O_3_.

The UV-vis diffuse reflectance spectra of different composites were exhibited in Fig. 4. It is obvious that the composite of TCB-30% showed a significant red shift of band edge to 456.5 nm compared with the initial TiO_2_, Bi_2_O_3_ and g-C_3_N_4_. There is also an intensively long tailing absorption in visible region, which can be explained by the synergistic effects of Bi_2_O_3_ and g-C_3_N_4_ quantum dots with a narrower band gap.

The band gap energies were determined using the equation below:*αhv* = *A*(*hv* − *E*_*g*_)^*n*/2^where *A*, *h*, *v*, *E*_*g*_ and *α* are the absorption coefficient, Planck's constant, frequency, band gap energy and a constant, respectively. For the composites in this study, *n* is 4 because of the indirect transition. Therefore, Tauc's plots of (*αhν*)^1/2^*versus* photon energy (*hν*) are obtained. As shown in Fig. 4b, the band gap energies of TiO_2_, TCB-30%, Bi_2_O_3_ and g-C_3_N_4_ are 3.20 eV, 2.24 eV, 2.80 eV and 2.7 eV, respectively. The TCB-30% has a lower band gap energy compared to TiO_2,_ which may be due to the heterojunction structure of the composite.

In order to further explain the photocatalytic activity of the ternary composite, the band structure of Bi_2_O_3_ was explored according to the following empirical equation.*E*_CB_ = *X* − *E*^e^ − 0.5*E*_*g*_*E*_CB_ = *E*_*VB*_ − *E*_*g*_where *E*_*VB*_ is the valence band edge potentials, E_CB_ is the conduction band energy, *X* is the electronegativity of semiconductor, *E*^e^ is the energy value of free electrons on the hydrogen scale, *E*_*g*_ is gap energy of semiconductor. The value of *X* for Bi_2_O_3_ is *ca.* 5.99 eV. The calculated conduction band and valence band of Bi_2_O_3_ are 0.33 eV and 3.13 eV, respectively. The band structure of TiO_2_ and g-C_3_N_4_ was calculated in above-mentioned method as well. And the results were showed in Table 1.

**Table 1:** Band structure of Bi_2_O_3_, TiO_2_ and g-C_3_N_4_

Catalyst	Band gap (eV)	Valence band (VB)	Conduction band (CB)
TiO_2_	3.2	2.7	−0.5
g-C_3_N_4_	2.7	1.4	−1.3
TCB-30%	2.2		
Bi_2_O_3_	2.8	3.13	0.33

### 3.5 Photocatalytic activity

**Fig. 5:**
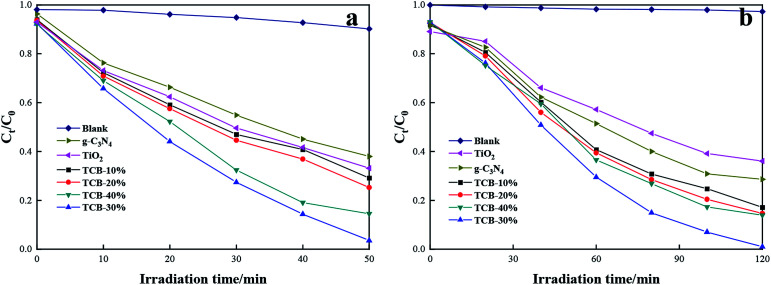
Photocatalytic activity of TCB-30%, TiO_2_ and g-C_3_N_4_ for degradation of RhB under (a) UV light irradiation (b) sunlight irradiation.

Fig. 5 displays the change of RhB concentration *versus* irradiation time with the pure TiO_2_, g-C_3_N_4_ or TCB-*x* under UV irradiation and sunlight irradiation. From Fig. 5a, pure TiO_2_ and pure g-C_3_N_4_ exhibited limited degradation rate of RhB under the UV light, which were only 66.9% and 62.1% after 50 min irradiation respectively. With the incorporation of Bi_2_O_3_ and construction of heterojunction, the photocatalytic activity substantially enhanced. In particular, the TCB-30% displayed the highest degradation rate of 96.5%. Moreover, TCB-30% showed the best photodegradation ability on RhB (completely degraded within 120 min) under sunlight irradiation (about 640 W m^−2^).

**Fig. 6:**
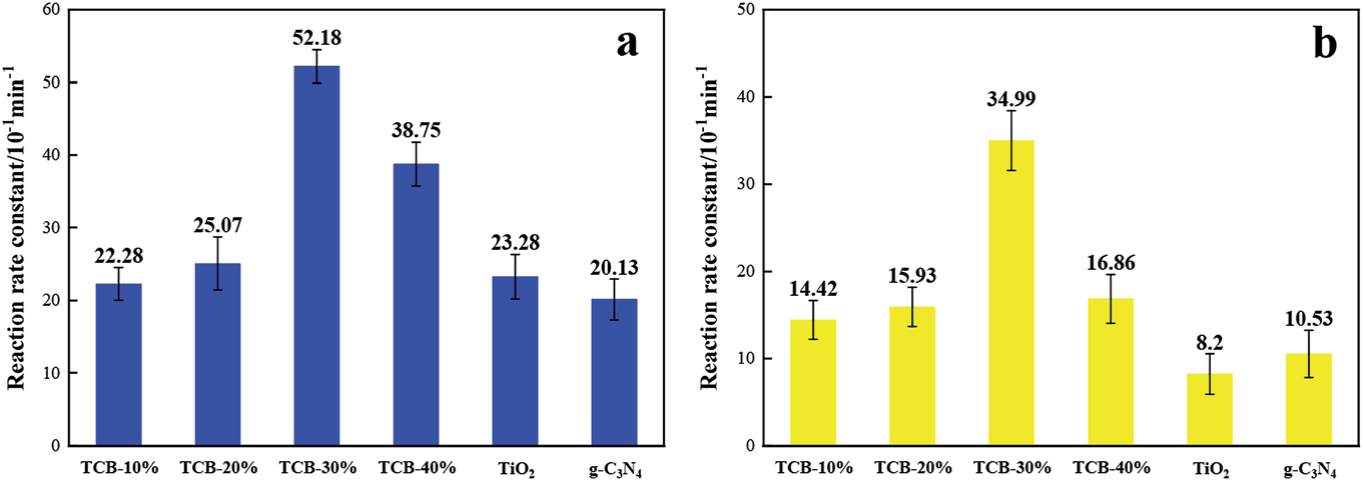
Reaction rate constant for degradation RhB of TCB-30%, TiO_2_, g-C_3_N_4_ and Bi_2_O_3_ under (a) UV light irradiation (b) sunlight irradiation.

The degradation of RhB followed the first-order kinetics, which can be expressed by the equation: ln(*C*_0_/*C*_*t*_) = *k*_a_^−1^*t*, where *C*_0_ and *C*_*t*_ is RhB concentration at time 0 and time *t*, and *k*_a_^−1^ represents the first-order reaction rate constant. As shown in Fig. 6, TCB-30% displayed a maximum value of rate constant under UV, which was about 2.2 times as high as that of TiO_2_ and 2.6 times as that of g-C_3_N_4_. When under the solar irradiation (640 W m^−2^), the *k*_a_^−1^ of TCB-30% was 4.2 times and 3.3 times higher than that of TiO_2_ and g-C_3_N_4_ respectively. Therefore, the improvement of photocatalytic activity under solar irradiation is more remarkable than that in the condition of UV light.

**Fig. 7:**
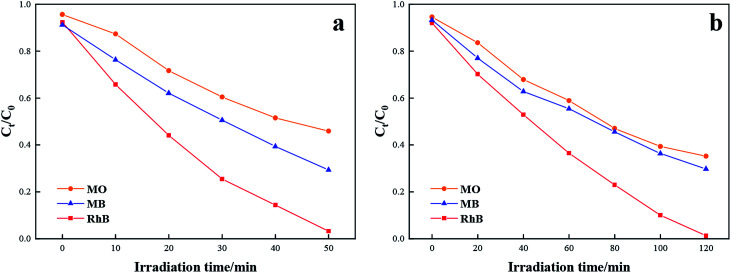
Comparison of the photocatalytic activity towards degradation of RhB, MB and MO under (a) UV light irradiation (b) sunlight irradiation.

**Fig. 8:**
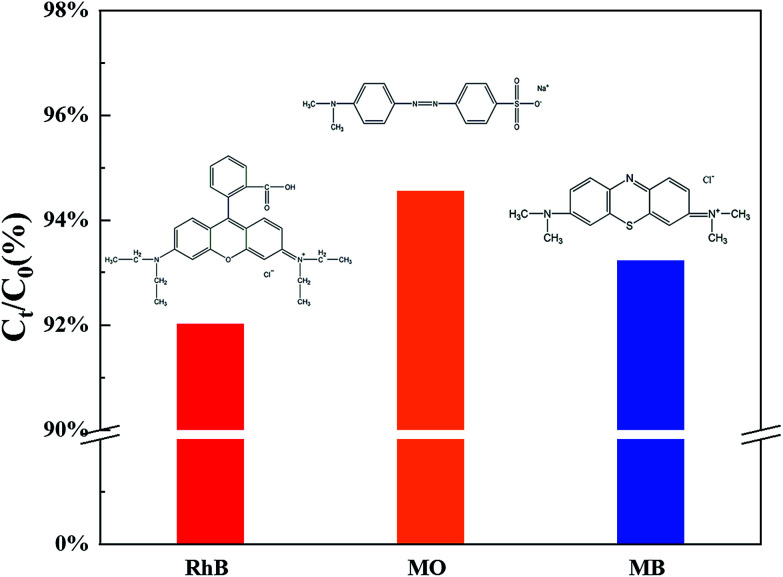
Dark adsorption of three dyes over TCB-30%.

Moreover, TCB-30% was also used to degrade different kinds of dyes with the same initial concentration under sunlight irradiation, including methylene blue (MB) and methyl orange (MO). As it shown in Fig. 7a, TCB-30% displayed a limited degradation rate for methylene blue and methyl orange compared with RhB. Fig. 8 showed the dark adsorption of three dyes over TCB-30%. It was supposed that the promising reaction rate could be contributed by the strong adsorption of the prepared heterojunction materials towards organic dyes with hydroxy groups. Thus, it is worth further research on how the surface groups of this composite affects the reaction with organic pollutants.

**Fig. 9:**
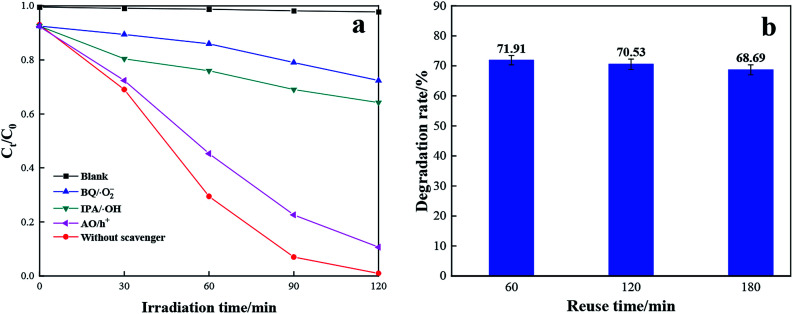
Radical capture test (a) and stability test of TCB-30% for degradation of RhB (b) under sunlight irradiation.

To figure out the contribution of photo-induced active radical in the reaction process, benzoquinone (BQ), isopropanol (IPA) and ammonium oxalate (AO) were applied as scavengers of superoxide radical (·O_2_^−^), hydroxyl radical (·OH) and hole (h^+^), respectively. The comparison experiments were carried out in the presence of the same molar concentration of scavengers (BQ, IPA and AO) for visible light degradation of RhB with TCB-30%. As displayed in Fig. 9, the degradation rate without any scavengers is 99.6%. By contrast, by adding BQ and IPA, the degradation rate sharply suppressed. While with the addition of AO scavengers, there is only a slight decrease of degradation rate. Therefore, it can be concluded that superoxide radical (·O_2_^−^) and hydroxyl radical (·OH) play key roles in degradation towards RhB under visible light.

During the practical application of catalysts, the by-products tend to be adsorbed on the active sites of photocatalysts' surface, leading to the dramatic decrease of the photocatalytic activity. To evaluate the stability of the prepared TCB-30%, recycle experiments were conducted under solar irradiation. As shown in the Fig. 9, the TCB-30% still presents the excellent photocatalytic performance after three cycles.

### 3.6 Mechanism discussion

**Fig. 10:**
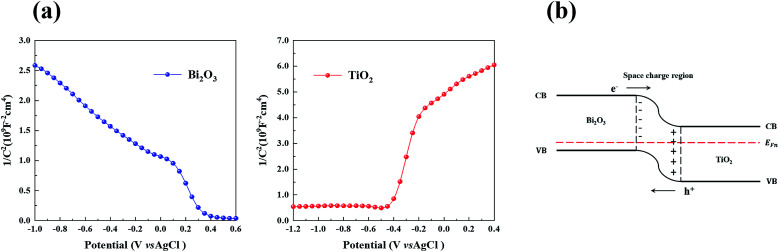
(a) Mott–Schottky plots of Bi_2_O_3_ and TiO_2_ in 0.5 M Na_2_SO_4_ at a frequency of 1 kHz, and (b) energy band structure of p-type Bi_2_O_3_/n-type TiO_2_.

In order to investigate the electrochemical properties of TiO_2_ and Bi_2_O_3_, Mott–Schottky plots were obtained. In Fig. 10a, TiO_2_ exhibit a positive slope indicating that it’s n-type semiconductor. On the contrary, BiO_2_ shows a negative slope, which indicts a p-type semiconductor.

**Scheme 1:**
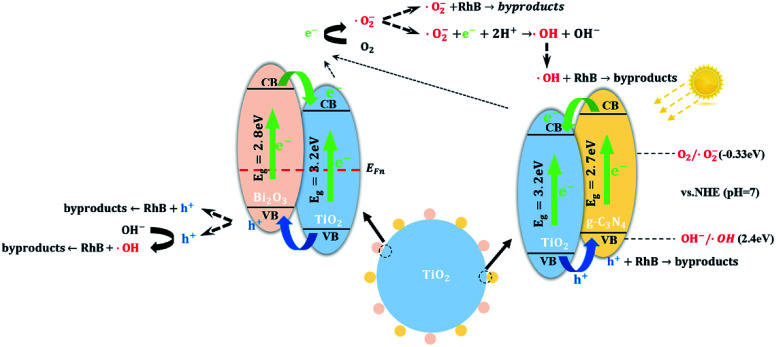
Photocatalytic degradation mechanism from the generation of photoinduced electron–hole to the decomposition of dyes.

Based on the results of XRD, XPS, TEM and UV-vis DRS analysis and Mott–Schottky, a tentative heterojunction system proposed and depicted in Scheme 1. TiO_2_ is placed as a support and connector of Bi_2_O_3_ and g-C_3_N_4_ quantum dots. The promising photocatalytic performance of TCB-30% under visible light is significantly related to the extended light adsorption spectrum and enhanced electron–hole separation.

On the one hand, the generation of g-C_3_N_4_ and Bi_2_O_3_ quantum dots with a narrow band gap facilitated the improvement of visible light harvesting ability, which motivates the generation of photoinduced carriers under visible light. On the other hand, the Fermi energy level of n-type semiconductors located nearer to the VB while the Fermi energy of p-type semiconductors located nearer to the VB.^23,27,35^ Therefore, after p-type Bi_2_O_3_ and n-type TiO_2_ were in contact, the electrons near the interface diffused from TiO_2_ to Bi_2_O_3_ while the holes diffused from Bi_2_O_3_ to TiO_2_. When the electrons migrated from TiO_2_, leaving a positively charged donor ion, and a positive charge region was formed on the side of the n region. In the same way, a negative charge region was formed on the side of p region. Therefore, there was no electrical neutrality on both sides of the interface of the p–n junction, and a positively and negatively charged region appeared, becoming a space charge region, which can force photoinduced electrons and holes to transfer in the opposite direction (Fig. 10b). The photogenerated electrons can only transfer from the CB of Bi_2_O_3_ to CB of TiO_2_, in contrast the holes can only transfer from the VB of TiO_2_ to the VB of Bi_2_O_3_, leading to a notably efficient separation of photoinduced pairs between Bi_2_O_3_ and TiO_2_. As for the heterojunction between g-C_3_N_4_ quantum dots and TiO_2_ particles, since the g-C_3_N_4_ has a more positive conduction band edge potential than that of TiO_2_ (−0.28 eV), the photoinduced electrons under visible irradiation tend to transfer from the CB of g-C_3_N_4_ to CB of TiO_2_.

Based on above calculation and analysis, an energy band structure has been proposed in Scheme 1. The photogenerated electrons and holes are unable to migrate from g-C_3_N_4_ to Bi_2_O_3_ through TiO_2_. Besides, the Bi_2_O_3_ and g-CN_4_ quantum dots don't interact and they are located in different position of TiO_2_ surface. Consequently, it's believed that there are just two heterojunctions (TiO_2_/g-C_3_N_4_, TiO_2_/Bi_2_O_3_) instead of a ternary heterojunction.

According to the previous researches, there are three types of radical degradation mechanism, including superoxide radical (·O_2_^−^), hydroxyl radical (·OH) and hole (h^+^).^3,7,8,44,45^ Steps of generation of the three radicals can be explained as follows:e^−^ + O_2_ → ·O_2_^−^·O_2_^−^ + H^+^ → HO_2_^˙^2HO_2_ → O_2_ + H_2_O_2_H_2_O_2_ +·O^−^_2_ → ·OH + OH^−^ + O_2_OH^−^ + h^+^ → ·OH

In this work, there was a minor change in degradation performance after eliminating the holes through the scavenging test. Thus, it was concluded that the majority dye was degraded by superoxide radical (·O_2_^−^) or hydroxyl radical (·OH) induced by photo catalysis. Meanwhile, a slight number of dyes is degraded by hydroxyl radical (·OH) induced by photoinduced holes or directly oxidation by the holes.

Eventually, the systematic mechanism, including the generation and transfer of carriers, generation of radicals and reaction between radicals and dyes, was proposed in Scheme 1.

## 4 Conclusion

In summary, a facile ball-milling/calcination was successively utilized to prepared a Bi_2_O_3_ and g-C_3_N_4_ quantum dots modified anatase TiO_2_ heterojunction system. It was found that TCB-30% exhibited the highest removal rate for degradation of dyes under UV light irradiation and sunlight irradiation. Under sunlight irradiation, there was remarkable improvement of TCB-30%, the reaction rate constant was about 4.2 times and 3.3 times higher than that of TiO_2_ and g-C_3_N_4_ towards degradation of RhB. The notable enhancement of photocatalytic activity is mainly due to the ability of visible light harvesting and efficient separation of the carriers, which was induced by the modification of Bi_2_O_3_ and g-C_3_N_4_ quantum dots.

## Conflicts of interest

There are no conflicts to declare.

## Supplementary Material
